# Central Nervous System Toxoplasmosis Beyond HIV in Patients with Hematologic Malignancies: A Contemporary Narrative Review and Illustrative Case Synthesis

**DOI:** 10.3390/ijms27209063

**Published:** 2026-10-13

**Authors:** Aleksandra Łanocha, Oliwia Pawelec-Pęciak, Karolina Kot, Bogusław Machaliński, Danuta Izabela Kosik-Bogacka, Natalia Łanocha-Arendarczyk

**Affiliations:** 1 Department of Hematology and Transplantology, Pomeranian Medical University in Szczecin, 70-252 Szczecin, Poland; aleksandra.lanocha@pum.edu.pl (A.Ł.); boguslaw.machalinski@pum.edu.pl (B.M.); 2 Department of Biology, Parasitology, and Pharmaceutical Botany, Pomeranian Medical University in Szczecin, Powstańców Wielkopolskich 72, 70-111 Szczecin, Poland; oliwia.pawelec.peciak@pum.edu.pl (O.P.-P.); karolina.kot@pum.edu.pl (K.K.); danuta.kosik.bogacka@pum.edu.pl (D.I.K.-B.)

**Keywords:** *Toxoplasma gondii*, central nervous system toxoplasmosis, toxoplasmic encephalitis, hematologic malignancies, hematopoietic stem cell transplantation, neuroimaging, molecular diagnosis, prophylaxis

## Abstract

Toxoplasmosis is an important opportunistic infection in immunocompromised individuals, typically resulting from reactivation of latent *Toxoplasma gondii* infection. CNS toxoplasmosis, most commonly presenting as toxoplasmic encephalitis (TE), is rare in patients with hematologic malignancies and after hematopoietic stem cell transplantation (HSCT). It is nevertheless life-threatening and likely underrecognized. This narrative review integrates molecular, immunological, clinical, and transplant-related evidence on *T. gondii* persistence and reactivation, treatment-associated immune dysfunction, diagnosis, management, prophylaxis, and molecular surveillance. Risk reflects cumulative immunosuppression, particularly impaired T-cell reconstitution, lymphocyte-depleting therapy, corticosteroid exposure, cytopenias, hypogammaglobulinemia, and absent or ineffective prophylaxis. Typical ring-enhancing lesions, positive serology, and detectable *T. gondii* DNA in cerebrospinal fluid may all be absent. Diagnosis therefore requires integration of immune and treatment history, magnetic resonance imaging, molecular testing, and, when necessary, histopathology. When clinical suspicion is high, empiric therapy should not be delayed; however, diagnostic specimens should be obtained whenever feasible, and evaluation for alternative diagnoses should proceed in parallel. In seropositive allogeneic HSCT recipients, trimethoprim–sulfamethoxazole prophylaxis with quantitative PCR surveillance remains the best-established preventive strategy.

## 1. Introduction

*Toxoplasma gondii* is a globally distributed intracellular protozoan capable of infecting humans and other warm-blooded animals, with seroprevalence varying markedly across geographical regions and populations [1,2,3,4]. Following primary infection, the parasite may establish lifelong latent infection, particularly within the central nervous system (CNS) and retina. In immunocompetent individuals, chronic infection is usually clinically silent. Impairment of cell-mediated immunity, however, may permit parasite reactivation and uncontrolled replication within neural tissue [2,5,6,7]. Historically, toxoplasmic encephalitis (TE) has been regarded primarily as an AIDS-defining opportunistic infection, particularly in patients with profound CD4+ T-cell depletion [3,6]. In the era of effective antiretroviral therapy, CNS toxoplasmosis is increasingly recognized in HIV-negative immunocompromised populations. These populations include patients with hematologic malignancies and recipients of hematopoietic stem cell transplantation (HSCT) [8,9,10,11]. In this review, CNS toxoplasmosis refers to any CNS involvement by the parasite, whereas TE denotes encephalitic brain involvement; the terms are not interchangeable. In patients with hematologic malignancies, diagnosis is complicated by nonspecific neurological manifestations, atypical neuroimaging, and false-negative serological or molecular tests [8,9,12,13]. Existing reviews and guidelines have largely focused on toxoplasmosis after HSCT, disseminated disease in hematologic populations, or opportunistic infections associated with contemporary anticancer therapies. A CNS-focused synthesis integrating treatment-related immune dysfunction with clinical, imaging, diagnostic, and management considerations remains limited. This review therefore examines CNS persistence and reactivation, clinically relevant risk profiles, clinical presentation, diagnosis, treatment, prophylaxis, and molecular monitoring in HIV-negative patients with hematologic malignancies.

## 2. Literature Search Approach

### 2.1. Information Sources and Search Strategy

This article was conceived as a narrative clinical review rather than a systematic review. PubMed/MEDLINE, Scopus, and Web of Science were searched from database inception through 23 July 2026. The search was restricted to English-language publications. Search terms were adapted to the specific topics addressed in the review and included *Toxoplasma gondii*, toxoplasmic encephalitis, cerebral toxoplasmosis, central nervous system (CNS) toxoplasmosis, hematologic malignancies, hematopoietic stem cell transplantation (HSCT), immunosuppressive therapies, neuroimaging, diagnosis, treatment, prophylaxis, reactivation, and molecular monitoring. Representative Boolean combinations included (“*Toxoplasma gondii*” OR “toxoplasmic encephalitis” OR “cerebral toxoplasmosis” OR “central nervous system toxoplasmosis”) AND (“hematologic malignancy” OR “hematological malignancy” OR leukemia OR lymphoma OR “multiple myeloma” OR “hematopoietic stem cell transplantation” OR HSCT), supplemented, where appropriate, by terms related to PCR, neuroimaging, diagnosis, treatment, prophylaxis, reactivation, and molecular surveillance. Reference lists of relevant primary studies, reviews, guidelines, and consensus documents were also screened to identify additional pertinent publications.

### 2.2. Eligibility, Screening, and Evidence Selection

The evidence base included clinical and cohort studies, case series, case reports, transplant surveillance studies, experimental and translational studies, clinical guidelines and consensus documents, and narrative or systematic reviews. These sources were included when they provided relevant information on CNS invasion and parasite persistence, treatment-related immune dysfunction, clinical presentation, neuropathology, neuroimaging, diagnosis, treatment, prophylaxis, molecular surveillance, or clinical outcomes. Titles and abstracts were independently screened for relevance by two authors (A.Ł. and N.Ł.-A.), followed by full-text assessment of potentially relevant publications. Any uncertainties regarding eligibility were resolved by discussion and consensus. Articles that did not address CNS toxoplasmosis or did not provide clinically or mechanistically relevant information were excluded. Where multiple publications described the same patient, cohort, or clinical episode, the most complete and informative report was preferentially used. Peer-reviewed literature was prioritized. Evidence from HIV-positive populations was retained selectively when it provided well-established mechanistic, diagnostic, neuroimaging, or therapeutic principles considered applicable to HIV-negative immunocompromised patients.

### 2.3. Evidence Hierarchy and Illustrative Case Selection

For questions concerning epidemiology, risk factors, diagnostic performance, prophylaxis, treatment, and clinical outcomes, greater interpretative weight was assigned to cohort studies, case series, transplant surveillance studies, clinical guidelines, and systematic or quantitative reviews than to isolated case reports. Individual case reports were used primarily to illustrate uncommon clinical or radiological presentations, diagnostic pitfalls, atypical disease courses, and challenges in clinical management. The reports summarized in Table 1 were selected as an illustrative clinical case synthesis rather than as a systematic case series. Eligible reports described HIV-negative patients with hematologic malignancies and/or HSCT who developed CNS toxoplasmosis and provided sufficient patient-level information on the underlying hematologic disorder, immunosuppressive treatment or transplantation status, clinical and/or radiological presentation, diagnostic approach, treatment, and outcome. Cases were selected to illustrate distinct clinical phenotypes, neuroimaging patterns, diagnostic approaches and pitfalls, therapeutic strategies, and outcomes. Duplicate reports, HIV-associated cases, reports with insufficient clinical information, and cases in which CNS involvement by *T. gondii* was not adequately supported were excluded. Table 1 was restricted to peer-reviewed published reports. The included cases were not used to estimate incidence, comparative risk, prognosis, diagnostic accuracy, or treatment effect. Consistent with the narrative design of the review, study selection did not follow a formal PRISMA-based systematic-review workflow; no protocol was prospectively registered, and no formal risk-of-bias assessment was performed.

### 2.4. Diagnostic Certainty and Disease Extent

Diagnostic certainty for the cases included in Table 1 and Supplementary Table S1 was classified as proven, probable, or possible CNS toxoplasmosis using an operational framework adapted from established definitions and current recommendations [20,21]. Proven CNS toxoplasmosis required direct histopathological or cytological demonstration of *Toxoplasma gondii* in CNS tissue. Probable CNS toxoplasmosis was defined by a compatible clinical and neuroimaging presentation supported by microbiological or molecular evidence, most commonly detection of *T. gondii* DNA by PCR in cerebrospinal fluid. Possible CNS toxoplasmosis referred to clinically and radiologically compatible cases lacking direct microbiological or histopathological confirmation and without an alternative diagnosis that adequately explained the findings. Response to specific anti-*Toxoplasma* therapy was considered supportive, but not confirmatory, evidence. Diagnostic certainty was assessed separately from disease extent. Cases were classified as CNS-only toxoplasmosis or disseminated toxoplasmosis with CNS involvement according to the presence of documented extracerebral disease. Isolated *T. gondii* DNAemia was not considered sufficient evidence of disseminated disease.

## 3. Biological Basis of CNS Persistence and Reactivation

The gastrointestinal tract represents the principal portal of entry for *Toxoplasma gondii*. After crossing the intestinal epithelium, rapidly proliferating tachyzoites disseminate systemically and may reach protected anatomical sites, including the CNS, retina, and placenta [2,22]. Several non-mutually exclusive mechanisms have been proposed to explain CNS invasion. These include direct interaction with cerebral endothelial cells, paracellular migration through endothelial tight junctions, transcellular passage across the blood–brain barrier (BBB), and leukocyte-mediated transport through the so-called “Trojan horse” mechanism, in which infected monocytes or other myeloid cells carry intracellular parasites into neural tissue [2,23,24]. Experimental studies suggest that infected monocytes may facilitate more efficient cerebral parasite accumulation than extracellular tachyzoites, supporting the biological relevance of leukocyte-mediated dissemination [25,26]. Cerebral capillaries, meningeal interfaces, and the choroid plexus may all serve as potential entry sites into the CNS [27].

Within neural tissue, tachyzoites differentiate into slowly replicating bradyzoites and establish persistent intracellular tissue cysts, predominantly in neurons, although infection of astrocytes and other glial cells has also been described [23,28]. These cysts may persist for the lifetime of the host and are relatively protected from both immune elimination and currently available antiparasitic drugs. CNS latency is therefore not a biologically inactive state but a dynamic equilibrium between parasite persistence and continuous host immune surveillance [29]. Tachyzoite-to-bradyzoite conversion is regulated by transcriptional and epigenetic mechanisms and may be influenced by cellular stress, host–cell type, nutrient availability, and inflammatory signals. Conversely, loss of immune control may promote bradyzoite-to-tachyzoite conversion, renewed intracellular replication, and progressive tissue injury [30]. Control of chronic CNS infection depends predominantly on cell-mediated immunity organized around the IL-12–IFN-γ axis [2,31,32]. During early infection, macrophages, dendritic cells, and neutrophils produce pro-inflammatory mediators, including IL-12, IL-18, and TNF-α, which promote IFN-γ production by NK cells and T lymphocytes [31,33]. IFN-γ enhances the recruitment and activation of CD4+ and CD8+ T cells and induces intracellular mechanisms that restrict parasite replication [34]. In murine models, these mechanisms prominently involve immunity-related GTPases and guanylate-binding proteins [35]. In human cells, IFN-γ-mediated control also includes indoleamine 2,3-dioxygenase 1-dependent tryptophan depletion, highlighting important species-specific differences that limit direct extrapolation from experimental models [36,37]. CD8+ T cells contribute through cytokine production and cytotoxic, including perforin-dependent, mechanisms, whereas microglia and astrocytes produce cytokines and chemokines that help maintain local parasite control [38]. At the same time, excessive inflammation may damage neural tissue [33]. The outcome of CNS infection therefore depends on a regulated balance between parasite restriction and immune-mediated injury. Counter-regulatory mediators, including IL-10, IL-4, and TGF-β, limit excessive inflammatory responses and reduce immunopathology. However, insufficient pro-inflammatory signaling or excessive immune suppression may favor parasite persistence and, under conditions of profound immunodeficiency, reactivation [33]. *T. gondii* may further influence this balance by modifying chemokine expression, macrophage activation, intracellular survival pathways, neuronal–microglial communication, and apoptosis. These mechanisms support long-term persistence in the CNS but may contribute to uncontrolled recrudescence when cellular immune surveillance becomes severely impaired [39,40,41].

At the molecular level, stage conversion is governed by dedicated parasite regulators. The Myb-like transcription factor BFD1 functions as a master regulator of bradyzoite differentiation and is required for efficient tissue-cyst formation, while ApiAP2 transcription factors and chromatin-based mechanisms further shape stage-specific gene expression [28,30]. *T. gondii* also actively counteracts IFN-γ-dependent cell-autonomous immunity. The dense-granule effector TgIST suppresses STAT1-dependent transcription and antagonizes IFN-γ-induced restriction in human cells, whereas polymorphic rhoptry effectors, including ROP5, ROP18, and ROP17, interfere with IRG/GBP-mediated attack on the parasitophorous vacuole in murine cells [34,35,36]. These observations highlight an important species-specific distinction: mice possess an expanded IFN-inducible IRG repertoire, whereas humans rely on different, strongly cell-type-dependent restriction mechanisms, including IDO1-mediated tryptophan depletion and GBP-associated or ubiquitin/autophagy-related responses [34,35,36,37]. Parasite genotype further modifies these interactions; strain-specific polymorphisms in secreted ROP and GRA effectors can alter host signaling and experimental virulence, although the extent to which genotype independently determines the phenotype or severity of human CNS toxoplasmosis remains uncertain [34,35].

**Figure 1:**
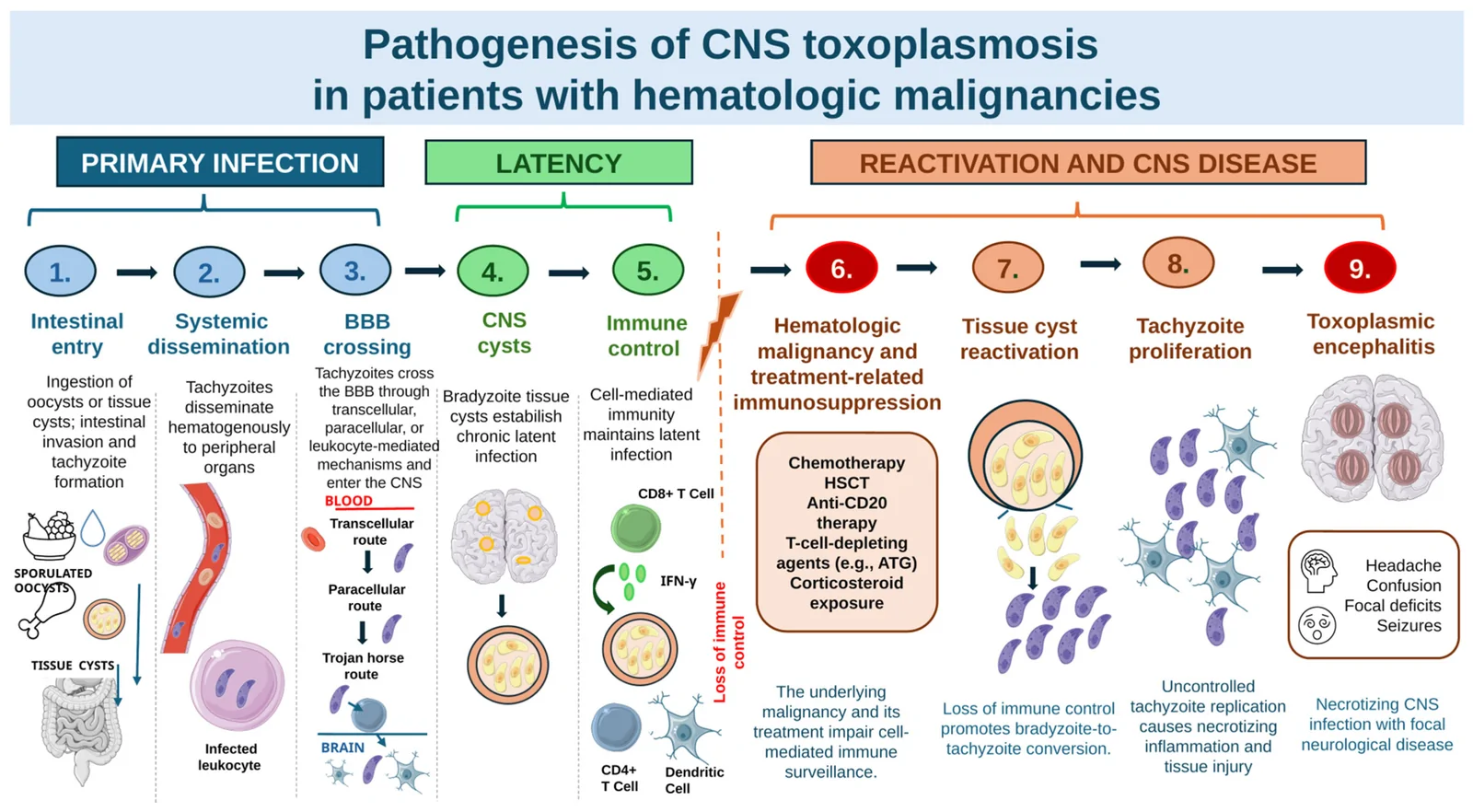
Stepwise pathogenesis of CNS toxoplasmosis in patients with hematologic malignancies. Following primary intestinal infection, tachyzoites disseminate through the bloodstream and may reach the CNS via endothelial, paracellular, transcellular, or leukocyte-mediated mechanisms. In neural tissue, bradyzoite-containing cysts persist under the control of cell-mediated immune surveillance. Hematologic malignancy and treatment-related immune dysfunction may impair this control, permitting reactivation of latent tissue cysts, bradyzoite-to-tachyzoite conversion, tachyzoite proliferation, and subsequent CNS tissue injury. The numbered circles (1–9) indicate successive stages of pathogenesis. Blue represents primary infection (1–3), green represents latency (4–5), and orange/red represents reactivation and CNS disease (6–9). Black arrows indicate the progression between stages, while the vertical dashed line and lightning symbol indicate the loss of immune control associated with parasite reactivation. BBB, blood–brain barrier; CNS, central nervous system; HSCT, hematopoietic stem cell transplantation; ATG, anti-thymocyte globulin. The schematic was created using Microsoft PowerPoint LTSC MSO 2021 (Version 16.0.14334.20756, 64-bit; Microsoft Corporation, Redmond, WA, USA). Selected graphical elements were adapted from Servier Medical Art and are licensed under CC BY 4.0.

In patients with hematologic malignancies, this equilibrium may be disrupted by both the underlying disease and treatment-related immune dysfunction. Prolonged lymphopenia, qualitative T-cell defects, B-cell depletion, hypogammaglobulinemia, corticosteroid exposure, lymphocyte-depleting therapies, and delayed immune reconstitution after hematopoietic stem cell transplantation may weaken the surveillance required to maintain latent cysts [42]. In seropositive allogeneic transplant recipients, most post-transplant cases are considered to result from reactivation during profound and sustained T-cell dysfunction, particularly when effective prophylaxis is absent, interrupted, delayed, or ineffective [20,43]. Loss of immune control permits bradyzoite-to-tachyzoite conversion and renewed parasite replication, leading to necrotizing inflammation, vascular injury, and focal or diffuse CNS disease [44]. This biological sequence provides the rationale for considering CNS toxoplasmosis in HIV-negative patients with hematologic malignancies who develop new neurological symptoms or unexplained CNS abnormalities. The sequence from primary intestinal infection through CNS latency and subsequent reactivation is summarized in Figure 1. These convergent defects, particularly impaired T-cell reconstitution and weakened IFN-γ-dependent parasite control, provide the mechanistic link between the net state of immunosuppression and reactivation of latent CNS cysts.

## 4. Net State of Immunosuppression and Treatment-Related Risk

Patients with hematologic malignancies constitute a biologically heterogeneous population in whom susceptibility to CNS toxoplasmosis reflects the combined effects of disease- and treatment-related immune dysfunction [11]. Bone-marrow failure, lymphopenia, neutropenia, hypogammaglobulinemia, and qualitative T-cell defects may be present before treatment and may persist or worsen over successive lines of therapy. The available evidence therefore supports assessment of the cumulative, or net, state of immunosuppression rather than attribution of risk to any single therapeutic agent [8,10,12,14].

### 4.1. Hematopoietic Stem Cell Transplantation

Allogeneic hematopoietic stem cell transplantation represents the best-established non-HIV setting for *T. gondii* reactivation. A systematic review of 46 studies comprising 47 datasets and 38,751 HSCT recipients identified 399 cases of toxoplasmosis, with a median reported prevalence of 2.14% overall and 7.51% among seropositive recipients [45]. In the landmark multicenter study conducted by the Infectious Diseases Working Party of the European Society for Blood and Marrow Transplantation (EBMT-IDWP), most affected patients were seropositive before transplantation, while acute graft-versus-host disease and the absence of *Toxoplasma*-active prophylaxis were common among patients who subsequently developed toxoplasmosis [21]. More recent surveillance studies further support associations between post-transplant reactivation and recipient seropositivity, acute graft-versus-host disease, conditioning intensity, corticosteroid exposure, and absent or interrupted prophylaxis [46]. Delayed T-cell reconstitution and prolonged immunosuppression may further extend the period of susceptibility, during which CNS toxoplasmosis may present either as isolated cerebral disease or as a manifestation of disseminated infection.

### 4.2. Purine Analogues and Other Lymphocyte-Depleting Therapies

Purine analogues, particularly fludarabine, induce profound and prolonged T-cell depletion that may persist after treatment discontinuation. CNS toxoplasmosis has been reported following fludarabine-containing regimens, including fludarabine–cyclophosphamide–rituximab (FCR), while anti-CD52 therapy with alemtuzumab causes sustained lymphocyte depletion and has also been associated with *T. gondii* reactivation, particularly in transplantation-related settings [8,14,47,48]. More recent case reports have described CNS toxoplasmosis following cladribine exposure; however, these observations remain hypothesis-generating and do not establish a causal relationship with cladribine itself [19].

### 4.3. B-Cell-Directed Treatment

Anti-CD20 monoclonal antibodies, including rituximab and obinutuzumab, cause prolonged B-cell depletion, hypogammaglobulinemia, and impaired humoral immune responses and may reduce the reliability of serological testing. Because control of latent *T. gondii* infection depends predominantly on cellular immunity, anti-CD20 exposure should be interpreted as one component of a broader net state of immunosuppression rather than an established independent risk factor for CNS toxoplasmosis. Reported cases following rituximab–cyclophosphamide–doxorubicin–vincristine–prednisone (R-CHOP) or obinutuzumab–chlorambucil generally involved additional treatment- or disease-related immune abnormalities [9,13].

### 4.4. CAR-T-Cell Therapy and Other Contemporary Treatments

CNS toxoplasmosis, including cerebral disease and disseminated toxoplasmosis with CNS involvement, has been reported after CAR-T-cell therapy, including cases initially misdiagnosed as immune effector cell-associated neurotoxicity syndrome (ICANS). These reports highlight CNS toxoplasmosis as an important differential diagnosis in patients who develop neurological deterioration after CAR-T therapy but do not establish CAR-T therapy as an independent risk factor. Affected patients are typically heavily pretreated and may have undergone prior HSCT or received lymphodepleting chemotherapy, prolonged corticosteroid therapy, or additional immunosuppression for treatment-related complications, often in the setting of prolonged cytopenias [49,50,51,52]. Cases have also been reported during prolonged lenalidomide maintenance and pomalidomide–dexamethasone-based therapy, further emphasizing the potential importance of cumulative treatment exposure and persistent immune dysfunction [19].

### 4.5. Illustrative Case-Based Synthesis

The reports summarized in Table 1 illustrate a heterogeneous spectrum of B- and T-cell lymphoid neoplasms, acute leukemias and multiple myeloma [53]. Most patients had received recent or prolonged antineoplastic therapy, undergone HSCT, or had persistent lymphopenia, neutropenia, pancytopenia, or hypogammaglobulinemia. Reports of CNS toxoplasmosis in patients with untreated hairy cell leukemia suggest that disease-related immune dysfunction alone may occasionally be sufficient to permit reactivation. Because the cases were selected for illustrative purposes, Table 1 should be interpreted as a qualitative synthesis of recurring clinical, radiological, diagnostic, therapeutic, and outcome patterns rather than as a comprehensive enumeration of published cases. Detailed patient-level data are provided in Supplementary Table S1.

## 5. Clinical Presentation and Red Flags

The clinical presentation of CNS toxoplasmosis in patients with hematologic malignancies is heterogeneous and frequently nonspecific, typically evolving subacutely over days to weeks. In the selected cases summarized in Table 1, common manifestations included altered mental status, focal neurological deficits, seizures, headache, aphasia or dysarthria, gait disturbance, and ataxia. Less typical presentations included visual-field defects, diplopia, peripheral facial nerve palsy, psychiatric symptoms, and spinal cord-predominant disease. Fever was inconsistently present and lacked diagnostic specificity. The differential diagnosis includes CNS relapse of the underlying malignancy, progressive multifocal leukoencephalopathy, treatment-related neurotoxicity, vascular complications, and other opportunistic infections. This clinical overlap may delay recognition, and several reported cases were confirmed only by biopsy or at autopsy. Practical clinical and risk-related red flags are summarized in Box 1 [8,9,10,12,14,15,19,47,54,55]. The absence of fever, meningeal signs, marked cerebrospinal fluid abnormalities, or a typical ring-enhancing pattern should not delay further evaluation in patients with profound cellular immune dysfunction.

Box 1 **Clinical and risk-related clues suggesting CNS toxoplasmosis in patients with hematologic malignancies.**

**Clinical clues** • altered mental status or otherwise unexplained cognitive deterioration; • new focal neurological deficits, aphasia, dysarthria, or cranial neuropathy; • seizures, gait disturbance, or ataxia; • multifocal neurological involvement or rapid deterioration of consciousness; • neurological or radiological progression despite corticosteroid therapy or treatment directed against presumed CNS involvement by the underlying malignancy.
**Risk-related clues** • documented pre-treatment or pre-transplant anti-*T. gondii* IgG seropositivity; • previous allogeneic HSCT, or autologous HSCT in the setting of additional persistent immune dysfunction; • recent or prolonged lymphocyte-depleting treatment, including purine analogues or alemtuzumab; • multiple previous lines of therapy, prolonged corticosteroid exposure, or delayed immune reconstitution; • persistent lymphopenia, severe CD4+ lymphopenia, neutropenia, other prolonged cytopenias, or hypogammaglobulinemia; • absence, interruption, or possible failure of *Toxoplasma*-active prophylaxis.
**Important caution**. The absence of fever, meningeal signs, marked CSF abnormalities, typical ring enhancement, or a positive CSF *T. gondii* PCR result does not exclude CNS toxoplasmosis.

## 6. Neuropathological Manifestations of CNS Toxoplasmosis

In severely immunocompromised patients, parenchymal CNS toxoplasmosis most commonly manifests as TE, characterized pathologically by progressive necrotizing inflammation. Two partially overlapping neuropathological patterns may occur. The first is an abscess-like necrotizing pattern associated with reactivation of latent tissue cysts and renewed tachyzoite proliferation. Lesions preferentially involve the gray–white matter junction, basal ganglia, and thalamus, although the brainstem, cerebellum, and corpus callosum may also be affected [3,6]. Histologically, an abscess-like focus may contain a central necrotic zone surrounded by an inflammatory rim with intra- and extracellular tachyzoites and variable numbers of residual tissue cysts [8,10,47,56,57]. The second pattern is predominantly vascular and reflects parasite invasion of endothelial or perivascular cells, with proliferative or necrotizing vasculitis, luminal occlusion, thrombosis, hemorrhage, and secondary infarction [57,58].

Parasites may be identified within vessel walls or vascular lumina rather than in adjacent infarcted parenchyma, emphasizing the contribution of vascular injury to disease progression. A recent histologically confirmed case demonstrated necrotizing encephalitis with obliterative angiopathy and both tachyzoites and bradyzoites detected by *T. gondii*-specific immunohistochemistry; the initial biopsy was interpreted as nonspecific, and the diagnosis required targeted re-evaluation [19]. Histopathological differentiation from primary CNS lymphoma, secondary leukemic or lymphomatous involvement, cerebral tuberculoma, fungal abscess, metastatic disease, and other CNS tumors may be challenging. Toxoplasmosis may also coexist with another opportunistic infection or neoplastic process, further complicating histopathological interpretation.

## 7. Neuroimaging Phenotypes and Differential Diagnosis

Neuroimaging is central to the initial evaluation of suspected CNS toxoplasmosis, but its interpretation is particularly challenging in patients with hematologic malignancies. In advanced HIV infection, TE classically presents as multiple ring-enhancing lesions with surrounding vasogenic edema and mass effect, commonly involving the basal ganglia, corticomedullary junction, and deep gray-matter structures [3,6]. In patients with hematologic malignancies or recipients of HSCT, neuroimaging may show a broader spectrum of findings, including solitary lesions, weak or absent contrast enhancement, hemorrhagic lesions, diffuse white-matter abnormalities, spinal cord involvement, and lymphoma-like masses [8,9,11,12,15,45]. Multiple lesions with surrounding edema predominated among the cases summarized in Table 1, although solitary and initially nonspecific abnormalities were also observed.

The eccentric and concentric target signs are supportive but insensitive imaging findings. In a focused review of CNS toxoplasmosis associated with B-cell malignancies, the eccentric target sign was identified in only two of nine previously published cases with available MRI data, whereas the concentric target sign was not reported; in a recent histologically confirmed case, both signs were recognized on initial imaging only in retrospect [19]. Evidence derived from patients with HIV further suggests that the radiological phenotype may vary according to the degree of cellular immunosuppression [59], although direct extrapolation to hemato-oncological populations remains uncertain. Accordingly, the absence of typical ring enhancement or a target sign should not substantially reduce clinical suspicion in a profoundly immunocompromised patient with a compatible neurological syndrome. The principal competing diagnoses and context-sensitive features that modify the pretest probability of CNS toxoplasmosis are summarized in Table 2. These distinctions are probabilistic rather than definitive and should be integrated in conjunction with treatment history, immune status, microbiological testing, and short-interval reassessment [60,61,62].

## 8. Diagnostic Strategy and Major Pitfalls

Diagnosis requires integration of clinical, radiological, serological, molecular, and, in selected cases, histopathological evidence; no single test can confirm or exclude CNS toxoplasmosis in every patient [19]. Detection of *T. gondii* DNA by PCR in CSF is one of the most valuable confirmatory approaches, but sensitivity is variable. False-negative results may reflect low parasite burden, focal or compartmentalized infection, sampling time, or previous anti-*Toxoplasma* treatment. In the cases summarized in Table 1, biopsy or autopsy confirmed infection despite negative or nondiagnostic CSF studies. Blood PCR provides complementary evidence, particularly after allogeneic HSCT or in disseminated disease, but a negative result does not exclude isolated CNS infection [6,8,9,12].

The genomic target selected for PCR can materially affect analytical sensitivity. The *B1* gene is present in approximately 35 copies per genome, whereas the non-coding REP-529 repeat occurs at substantially higher copy number (approximately 200–300 copies), which generally increases analytical sensitivity when parasite DNA burden is low [1]. Comparative studies have therefore often favored REP-529 over *B1*; however, assay performance remains dependent on primer/probe design, DNA extraction, specimen type, parasite burden, and laboratory validation. Accordingly, the PCR target should be reported and considered when interpreting negative or low-level qPCR results [1].

Anti-*T. gondii* IgG identifies previous exposure and supports the possibility of reactivation but does not establish active disease. Conversely, negative serology may be unreliable in profound immune dysfunction, hypogammaglobulinemia, or after B-cell-depleting therapy. IgM is generally unhelpful in reactivation and occasional positivity may be misleading. Reports using targeted next-generation sequencing (tNGS) and plasma microbial cell-free DNA (mcfDNA) sequencing illustrate the potential value of advanced molecular methods when conventional testing is inconclusive [15,78,79]. These approaches remain adjunctive and should be described using method-specific terminology; they do not replace conventional microbiological, radiological, or pathological assessment.

**Figure 2:**
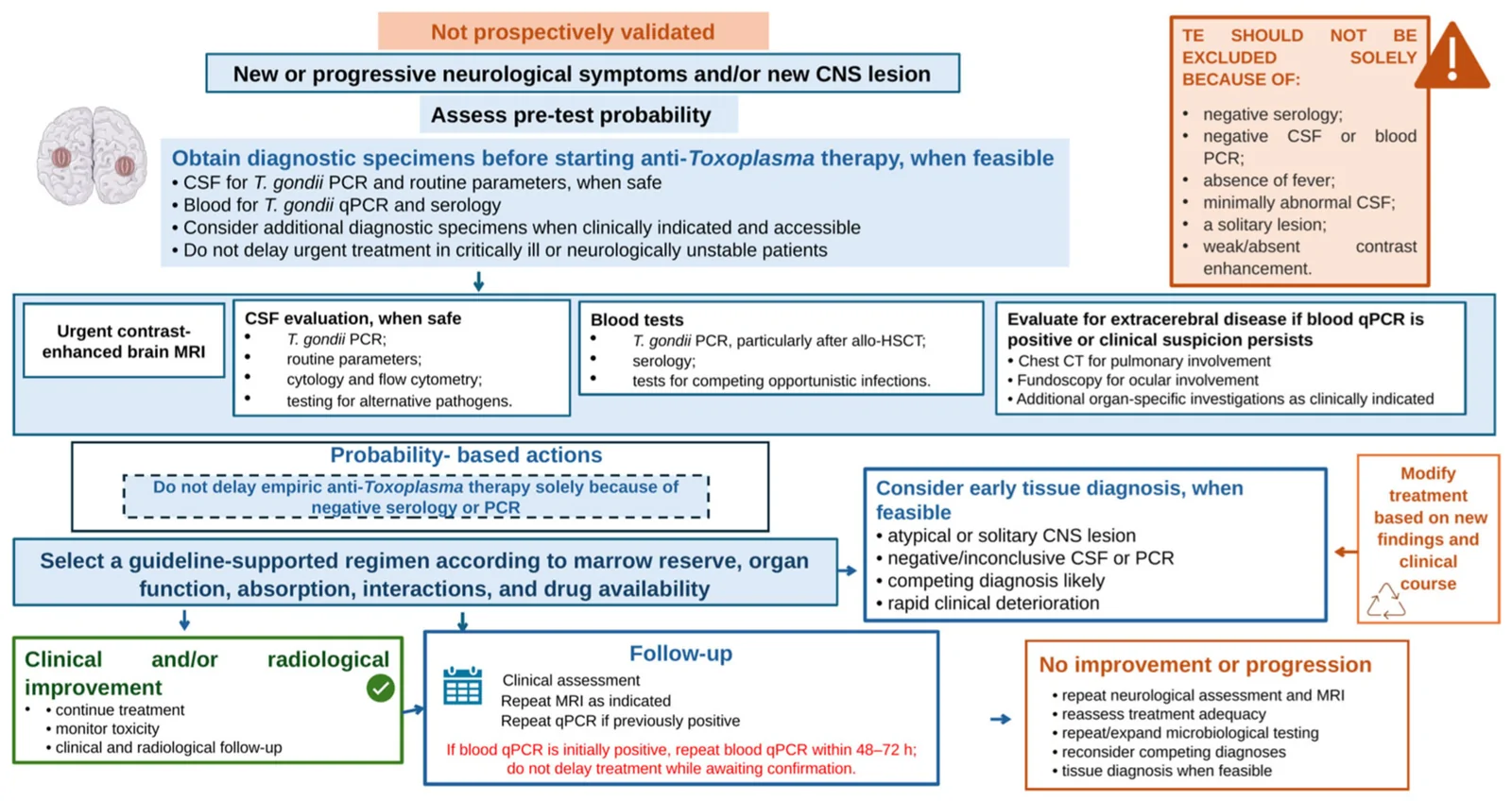
Expert-derived, evidence-informed diagnostic and management proposal for suspected CNS toxoplasmosis in hematologic patients. The algorithm summarizes a pragmatic approach to patients with new or progressive neurological symptoms and/or a new CNS lesion, integrating assessment of pretest probability, collection of diagnostic specimens before initiation of anti-*Toxoplasma* therapy when feasible, urgent neuroimaging, CSF and blood testing, evaluation for extracerebral disease, and probability-based initial management. The scheme also highlights repeat qPCR testing after an initial positive result, criteria supporting early tissue diagnosis, and a structured reassessment pathway when clinical or radiological improvement is absent. . Arrows indicate the direction of the proposed diagnostic and management pathway. Blue boxes represent diagnostic evaluation, management, and follow-up; green indicates clinical and/or radiological improvement; and orange indicates the need for reassessment or modification of management. Red text highlights important clinical cautions, while the dashed border emphasizes a key consideration regarding empiric therapy. The proposed algorithm has not been prospectively validated. The schematic was created in Microsoft PowerPoint LTSC MSO 2021 (Version 16.0.14334.20756, 64-bit; Microsoft Corporation, Redmond, WA, USA).

Tissue diagnosis should be considered promptly when lesions progress, the clinical course or imaging pattern is atypical, molecular tests remain negative, or competing diagnoses such as CNS lymphoma, invasive fungal infection, or treatment-related pathology cannot be excluded. Sampling error is possible; therefore, nondiagnostic tissue should be re-evaluated with parasite-specific immunohistochemistry and, where feasible, molecular testing when suspicion remains high [44]. A pragmatic probability-based diagnostic and initial-management pathway is proposed in Figure 2.

## 9. Treatment, Prophylaxis, and Molecular Monitoring

### 9.1. Empiric and Definitive Treatment

Management of CNS toxoplasmosis in patients with hematologic malignancies or following HSCT is complicated by cytopenias, organ dysfunction, polypharmacy, impaired enteral absorption, and ongoing treatment-related immunosuppression. Evidence specifically addressing established toxoplasmosis in these populations remains limited, and ECIL-9 recommendations are derived largely from experience with HIV-associated toxoplasmic encephalitis [20,80]. In patients with a high pretest probability of CNS toxoplasmosis based on the immunosuppressive context, clinical presentation, and neuroimaging findings, anti-*Toxoplasma* therapy should be initiated promptly; a negative blood PCR or serological result should not, in isolation, exclude the diagnosis. Empiric treatment should proceed in parallel with investigation for competing diagnoses, particularly CNS lymphoma, invasive fungal infection, bacterial or nocardial abscesses, and other opportunistic or neoplastic processes. Pyrimethamine combined with sulfadiazine and folinic acid remains a preferred regimen for established disease, whereas pyrimethamine–clindamycin is an alternative in patients unable to tolerate sulfadiazine. TMP–SMX, administered orally or intravenously at therapeutic doses, is an accepted alternative when pyrimethamine is unavailable or the oral route is not feasible, irrespective of prior TMP–SMX prophylaxis [20,80]. Treatment should be individualized according to neurological severity, marrow reserve, renal and hepatic function, gastrointestinal absorption, prior prophylaxis, and clinically relevant drug interactions.

### 9.2. Treatment, Duration, and Response Assessment

Established *Toxoplasma* disease should be treated for a minimum of 6 weeks, with longer courses considered in patients with extensive clinical or radiological disease or an incomplete response at 6 weeks [20]. Response assessment should integrate clinical, radiological, and molecular findings. ECIL-9 recommends documenting molecular clearance with two consecutive negative blood qPCR results obtained 7 days apart and, where applicable, at least one negative result from any other specimen that was previously qPCR-positive, including CSF [20]. Because parasitological clearance and clinical recovery may not occur simultaneously, qPCR kinetics should be interpreted in the broader clinical context. Persistent clinical or radiological deterioration despite appropriate therapy should prompt reassessment of drug exposure and absorption, treatment adequacy, and competing diagnoses, with tissue diagnosis considered when clinically appropriate. Reduction in immunosuppression should be pursued whenever feasible. Adjunctive corticosteroids are not routinely recommended and should be reserved for selected patients with severe mass effect, radiological midline shift, elevated intracranial pressure, or comparable neurological emergencies [20]. When required, corticosteroids should be administered only together with active antiparasitic treatment and at the lowest effective dose for the shortest possible duration.

### 9.3. Primary Prophylaxis, qPCR Surveillance, and Pre-Emptive Therapy

**Figure 3:**
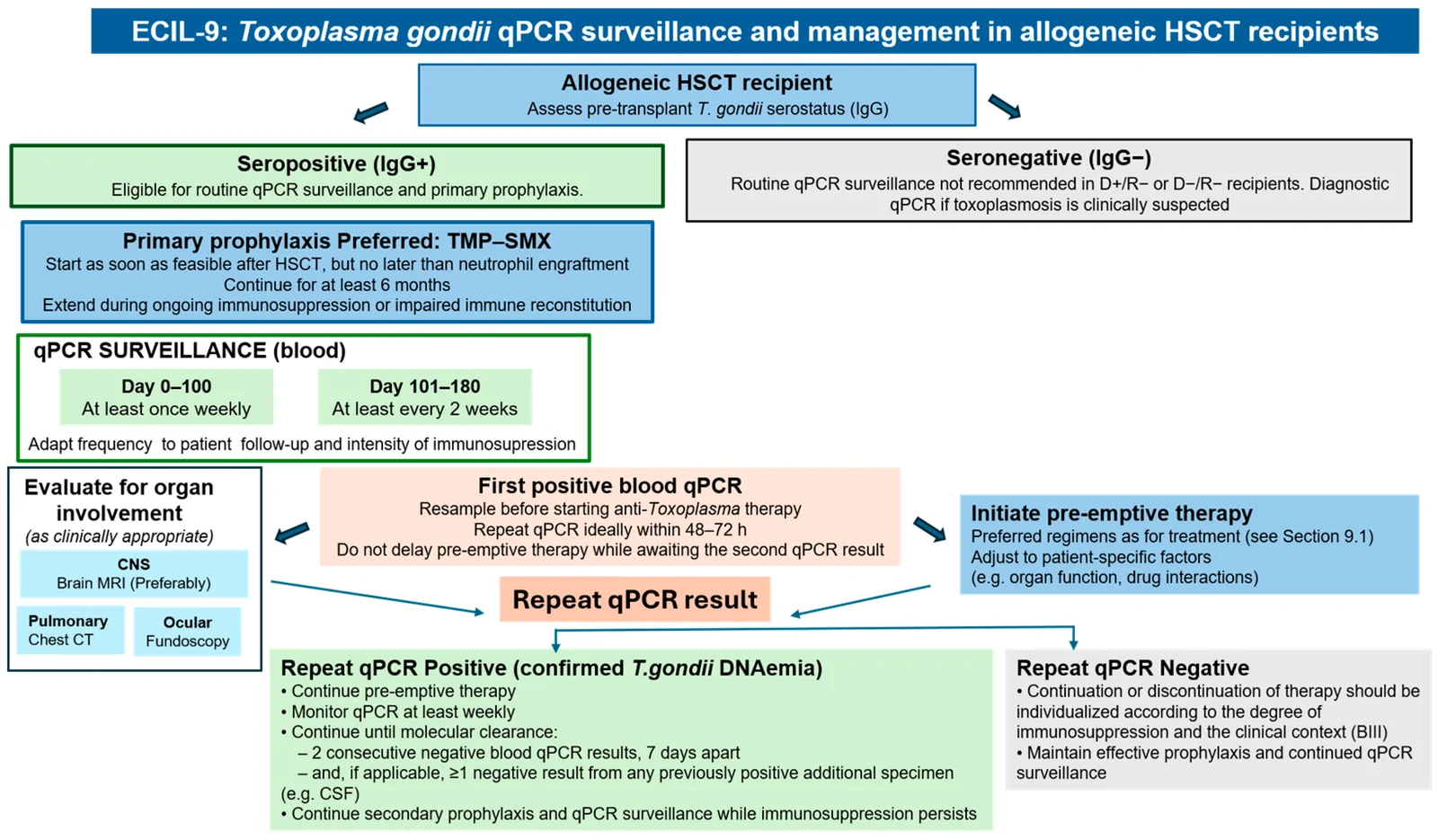
ECIL-9–based algorithm for *Toxoplasma gondii* qPCR surveillance and pre-emptive management in allogeneic HSCT recipients. In *T. gondii*-seropositive recipients, blood qPCR monitoring is recommended at least weekly through day 100 and every 2 weeks through day 180, with further surveillance adapted to ongoing immunosuppression. An initial positive result should prompt repeat testing within 48–72 h, preferably before treatment, without delaying therapy when clinically indicated, together with evaluation for CNS, pulmonary, and ocular involvement. Molecular clearance is defined by two consecutive negative blood qPCR results 7 days apart. Management of an isolated positive result followed by a negative repeat test should be individualized according to immunosuppression and clinical context (BIII). Arrows indicate the direction of the surveillance and management pathway. Blue boxes represent clinical management and diagnostic evaluation; green boxes highlight seropositivity, routine surveillance, and confirmed DNAemia; the pale orange box indicates an initial positive qPCR result; and gray boxes represent seronegative status or individualized management decisions. Adapted from the ECIL-9 recommendations [20]. The schematic was created in Microsoft PowerPoint LTSC MSO 2021 (Version 16.0.14334.20756, 64-bit; Microsoft Corporation, Redmond, WA, USA). **Abbreviations:** CNS, central nervous system; CSF, cerebrospinal fluid; CT, computed tomography; D, donor; D+/R−, seropositive donor/seronegative recipient; D−/R−, seronegative donor/seronegative recipient; ECIL, European Conference on Infections in Leukaemia; HSCT, hematopoietic stem cell transplantation; IgG, immunoglobulin G; MRI, magnetic resonance imaging; qPCR, quantitative polymerase chain reaction; R, recipient; TMP–SMX, trimethoprim–sulfamethoxazole; BIII, ECIL grade based primarily on expert opinion and descriptive evidence.

ECIL-9 recommends primary *T. gondii* prophylaxis for all seropositive allogeneic HSCT recipients, with TMP–SMX as the preferred agent. Prophylaxis should begin as soon as feasible after transplantation but no later than neutrophil engraftment; because of potential marrow toxicity, TMP–SMX is generally avoided during the pre-engraftment period. It should be continued for at least 6 months and extended during ongoing treatment-related immunosuppression or delayed immune reconstitution [20]. Blood qPCR surveillance is recommended in seropositive allogeneic HSCT recipients, including those receiving appropriately dosed TMP–SMX because breakthrough reactivation may occur. Screening should begin at transplantation, be performed at least weekly through day 100 and at least every 2 weeks through day 180, with frequency adapted to clinical follow-up and the intensity of immunosuppression [20]. After a first positive blood qPCR, a repeat specimen should ideally be obtained within 48–72 h and before anti-*Toxoplasma* treatment, without delaying therapy while awaiting confirmation. Patients should concurrently be evaluated for organ involvement, particularly CNS, pulmonary, and ocular disease, using brain imaging—preferably MRI—chest CT, and fundoscopy as clinically indicated [20]. During pre-emptive therapy, qPCR should be monitored at least weekly to assess parasite-load kinetics. If the repeat qPCR is negative, continuation or discontinuation of treatment should be individualized according to the degree of immunosuppression and the overall clinical context; if treatment is discontinued, effective prophylaxis and continued molecular surveillance should be maintained. When pre-emptive therapy is continued, treatment should proceed until two consecutive negative blood qPCR results have been obtained 7 days apart [20]. Figure 3 summarizes the ECIL-9–aligned approach to serial blood qPCR monitoring and pre-emptive management of *Toxoplasma gondii* reactivation in seropositive allogeneic HSCT recipients.

### 9.4. Secondary Prophylaxis

Following pre-emptive therapy, and after treatment of established toxoplasmosis in patients with persistent risk factors for recurrence, secondary prophylaxis should be considered together with continued qPCR surveillance while clinically relevant immunosuppression or low CD4 counts persist [20]. The choice and duration of prophylaxis should be individualized according to immune reconstitution, ongoing immunosuppressive therapy, prior prophylactic and therapeutic regimens, and treatment tolerance.

## 10. Geographical Context and International Applicability

The clinical context of CNS toxoplasmosis shows substantial geographic variation. Baseline exposure to *T. gondii* determines the proportion of hematologic patients at risk of reactivation and thereby influences the pretest probability of disease. Marked geographic heterogeneity has been documented worldwide, with particularly high pooled estimates reported in South America and Africa and substantial regional variation within Asia and Europe [1,81,82]. Considerable heterogeneity is also evident within Europe, with pooled estimates reaching approximately 50% in Eastern Europe, 48% in Western Europe, 45% in Southeastern Europe, and 38% in Southwestern Europe [82]. These epidemiological differences are clinically relevant when interpreting serological status, assessing reactivation risk, and determining the threshold for further diagnostic evaluation. Geographic variation also extends to the population structure of *T. gondii*. Type II lineages predominate in Europe, whereas South America is characterized by substantially greater genetic diversity, including atypical and recombinant genotypes; parasite populations in Africa and Asia exhibit distinct regional patterns and remain less comprehensively characterized in clinical hematology cohorts [83,84]. The clinical significance of this diversity in hematologic patients remains uncertain, although regional strain variation may contribute to differences in disease phenotype. The international applicability of diagnostic and therapeutic strategies is further influenced by disparities in laboratory infrastructure and drug availability. qPCR and other advanced molecular techniques are more accessible in well-resourced centers, whereas conventional or nested PCR may remain more widely used in resource-limited settings [85]. Similarly, limited access to pyrimethamine in some countries may increase reliance on TMP–SMX as an alternative regimen [80]. Diagnostic and therapeutic recommendations should therefore be interpreted in the context of local epidemiology, laboratory capacity, and access to antiparasitic therapy.

## 11. Limitations of the Evidence Base and Future Directions

The available evidence is dominated by individual case reports and small case series and is therefore vulnerable to publication bias, selective reporting, and overrepresentation of severe, atypical, or fatal presentations. Diagnostic certainty varied across reports, while immune status, CD4+ T-cell counts, serostatus, prophylaxis, timing and intensity of treatment exposure, drug dosing, and follow-up were inconsistently reported. Many patients had several concurrent risk factors, preventing reliable attribution of reactivation to a specific drug or treatment class. Because the case summary was assembled for illustrative purposes within a narrative review rather than through a systematic selection process, it should not be regarded as exhaustive. The absence of population denominators also precludes reliable estimation of incidence, comparative risk, prognosis, or treatment effectiveness from the presented reports. Future research should distinguish the established allogeneic HSCT setting from populations in which screening and preventive strategies remain uncertain. In seropositive allogeneic HSCT recipients, studies should refine the optimal duration of prophylaxis, molecular monitoring intervals, thresholds for pre-emptive treatment, management of breakthrough reactivation, and criteria for discontinuing prophylaxis after immune reconstitution. In nontransplant hematology patients, standard autologous HSCT recipients, and patients receiving CAR-T-cell therapy, prospective studies are needed to determine which risk profiles warrant baseline serological screening, serial qPCR surveillance, or primary prophylaxis. Particular attention should be given to patients with persistent lymphopenia or hypogammaglobulinemia and those receiving purine analogues, alemtuzumab, bendamustine, anti-CD20-based combinations, or multiple successive lines of immunosuppressive treatment. Advanced neuroimaging and molecular diagnostic methods, including tNGS and mcfDNA sequencing, require prospective evaluation in well-characterized cohorts. Studies should assess their diagnostic sensitivity and specificity, turnaround time, incremental value over conventional testing, potential for false-positive or clinically ambiguous results, and ability to accelerate appropriate treatment or reduce the need for brain biopsy. Multicenter registries with standardized definitions of diagnostic certainty, immune status, treatment exposure, prophylaxis, and outcomes could provide the denominators required to clarify the contemporary incidence, prognosis, and treatment-related risk of CNS toxoplasmosis. Future studies should translate these mechanistic and case-based observations into prospectively validated strategies for risk stratification, molecular monitoring, and targeted prophylaxis. The restriction of the literature search to English-language publications is an additional limitation and may have introduced language and geographical selection bias, potentially underrepresenting evidence from regions where toxoplasmosis is highly prevalent.

## 12. Conclusions

CNS toxoplasmosis should remain a key differential diagnosis in profoundly immunocompromised patients with hematologic malignancies who develop new or progressive neurological syndromes, even when serology, CSF qPCR, or neuroimaging findings are inconclusive. Diagnostic assessment should integrate the cumulative burden of immunosuppression, neuroimaging, molecular testing, and competing diagnoses, with early tissue diagnosis considered when uncertainty persists. In seropositive allogeneic HSCT recipients, TMP–SMX prophylaxis and qPCR surveillance remain the best-established preventive strategy, whereas evidence is insufficient to support routine surveillance in other hematologic populations. Prospective multicenter studies are needed to validate risk-stratification and diagnostic-management pathways.

## Figures and Tables

**Table 1:** Illustrative reports highlighting the clinical, radiological, and diagnostic heterogeneity of CNS toxoplasmosis in HIV-negative patients with hematologic malignancies. Cases were selected to illustrate distinct clinical presentations, neuroimaging patterns, diagnostic approaches and pitfalls, levels of diagnostic certainty, and disease extent. The table provides a qualitative clinical synthesis and was not intended as a systematic case series or as a basis for quantitative inference.

Age/Sex	Hematologic Setting	Key Clinical/Imaging Phenotype	Key Diagnostic Evidence	Diagnostic Category	Disease Extent	Source
63/F	Chronic lymphocytic leukemia, CLL	Gait instability, visual changes and rapid mental-status decline; multiple intracranial lesions	Brain biopsy demonstrated *T. gondii* tachyzoites	Proven	CNS-only	[10]
63/M	Chronic lymphocytic leukemia, CLL	Apraxia, forgetfulness and progressive cognitive impairment; frontal/parietal white-matter lesions	Brain biopsy showed ruptured *Toxoplasma* cysts; CSF PCR positive for *T. gondii*; concurrent JCV/PML	Proven	CNS-only	[14]
55/M	Acute T-cell leukemia, complete remission	Neurological symptoms during complete remission; focal cerebral disease	Stereotactic brain biopsy confirmed toxoplasmic encephalitis	Proven	CNS-only	[8]
5/F	Acute lymphoblastic leukemia, ALL	Fever during chemotherapy; multifocal cerebral and cerebellar MRI abnormalities	*T. gondii* sequences detected by tNGS in CSF and blood; blood real-time PCR negative	Probable	CNS-only	[15]
76/M	Relapsed/refractory IgA kappa multiple myeloma	Rapidly progressive altered mental status; numerous ring-enhancing lesions with eccentric target signs	CSF *T. gondii* PCR positive (57,900 copies/mL)	Probable	CNS-only	[16]
77/F	Diffuse large B-cell lymphoma, DLBCL in remission	Progressive right-sided weakness; multiple lesions in the basal ganglia and at the gray-white junction	CSF *T. gondii* PCR positive; post-mortem brain histology and immunohistochemistry positive for *T. gondii*	Proven	CNS-only	[17]
56/F	CLL transformed to high-grade non-Hodgkin lymphoma, NHL	Right-arm pain/paresthesia with enhancing C5/C6 spinal lesion; later pulmonary and systemic deterioration	Weakly positive CSF *T. gondii* PCR; autopsy/IHC demonstrated T. gondii in brain, spinal cord, heart and lungs	Proven	Disseminated with CNS involvement	[18]
61/F	Multiple myeloma in sustained remission	Right peripheral facial palsy and mild ptosis; multiple supra- and infratentorial enhancing lesions	CSF *T. gondii* PCR negative; no brain biopsy; latent *T. gondii* serology and therapeutic response were supportive only	Possible	CNS-only	[19]

**Note:** Diagnostic categories were assigned by the review authors according to the operational framework defined in Section 2.4 and may therefore differ from terminology used in the original reports. Proven CNS toxoplasmosis requires direct histopathological or cytological demonstration of *T. gondii* in CNS tissue. Probable CNS toxoplasmosis requires a compatible clinical and neuroimaging presentation supported by microbiological or molecular evidence. Possible CNS toxoplasmosis refers to compatible cases lacking direct microbiological or histopathological confirmation. A clinical and/or radiological response to anti-*Toxoplasma* therapy is considered supportive, but not confirmatory, evidence. Disease extent was assessed separately; isolated detection of *T. gondii* DNA in peripheral blood was not considered sufficient evidence of disseminated disease. **Abbreviations:** ALL, acute lymphoblastic leukemia; CLL, chronic lymphocytic leukemia; CNS, central nervous system; CSF, cerebrospinal fluid; DLBCL, diffuse large B-cell lymphoma; IHC, immunohistochemistry; JCV, JC virus; MRI, magnetic resonance imaging; NHL, non-Hodgkin lymphoma; PCR, polymerase chain reaction; PML, progressive multifocal leukoencephalopathy; tNGS, targeted next-generation sequencing.

**Table 2:** Focused differential diagnosis of suspected CNS toxoplasmosis in HIV-negative patients with hematologic malignancies.

Diagnostic Consideration	Clinical Relevance	Key features Favoring the Alternative Diagnosis	Features Increasing the Pretest Probability of CNS Toxoplasmosis
Primary CNS lymphoma, lymphoma relapse, or secondary CNS involvement by leukemia/lymphoma	The most important neoplastic mimic; it may be clinically indistinguishable from CNS toxoplasmosis at presentation.	A solitary deep, periventricular, or callosal lesion with homogeneous enhancement; marked diffusion restriction, increased perfusion, or high ^18^F-FDG uptake may favor lymphoma. Leptomeningeal or ependymal disease, systemic relapse, or progression despite appropriate anti-*Toxoplasma* therapy further support lymphoma. A corticosteroid response does not exclude PCNSL [63,64].	Compatible profound cellular immunosuppression, documented prior *T. gondii* exposure, absent or interrupted TMP–SMX prophylaxis, and no independent evidence of hematologic CNS relapse. Lower ^18^F-FDG uptake may support CNS toxoplasmosis in selected cases but is not diagnostic because uptake overlaps with lymphoma [63,64].
Fungal abscess or angioinvasive fungal CNS infection	Particularly relevant after HSCT, prolonged neutropenia, corticosteroid exposure, or intensive chemotherapy.	Pulmonary or sinus fungal disease, positive fungal biomarkers, hemorrhagic infarcts, vascular-territory lesions, or other evidence of angioinvasion [63,64,65,66].	Documented prior *T. gondii* exposure, absent or interrupted prophylaxis, compatible focal lesions, and no microbiological or radiological evidence of invasive fungal disease; none of these findings is diagnostic in isolation.
Progressive multifocal leukoencephalopathy	Consider after anti-CD20 therapy or other profound B-cell or T-cell immunosuppression.	Predominant asymmetric subcortical white-matter involvement, U-fiber involvement, minimal mass effect, and JC virus detection in CSF [14,66].	Ring-enhancing focal lesions with substantial edema or mass effect, basal ganglia involvement, and a compatible *T. gondii* risk profile.
Treatment-related neurotoxicity or vascular complications	An important noninfectious mimic during chemotherapy, immunotherapy, or transplantation-related treatment.	A close temporal relationship with the suspected therapy; a PRES pattern, vascular-territory ischemia or hemorrhage, hypertension, coagulopathy, or thrombocytopenia [52,66,67].	Enhancing focal lesions with edema, compatible neurological symptoms, prior *T. gondii* exposure, and absent TMP–SMX prophylaxis.
Cerebral tuberculoma or other mycobacterial CNS disease	Relevant with epidemiological exposure, latent tuberculosis, systemic symptoms, or profound immunosuppression.	Meningeal enhancement, hydrocephalus, pulmonary or disseminated tuberculosis, inflammatory CSF abnormalities, or microbiological evidence of mycobacterial infection [66].	A compatible *T. gondii* risk profile and focal enhancing lesions in the absence of epidemiological, systemic, CSF, or microbiological evidence of tuberculosis.
Viral encephalitis, particularly HSV or VZV	An important infectious mimic in immunocompromised patients; HSV may cause encephalitis, whereas VZV may cause meningoencephalitis or cerebral vasculopathy [66,68,69].	HSV encephalitis may preferentially involve temporal or limbic regions. VZV CNS disease may present as meningoencephalitis or cerebral vasculopathy with ischemic or hemorrhagic lesions. CSF viral qPCR supports the diagnosis in the appropriate clinical context, but a negative result does not invariably exclude disease, particularly after antiviral therapy has begun [69,70].	Multifocal enhancing lesions with marked edema or mass effect, basal ganglia involvement, a compatible *T. gondii* risk profile, and *T. gondii*-specific molecular evidence increase the probability of CNS toxoplasmosis.
HHV-6B or CMV CNS disease	Particularly relevant after allogeneic HSCT and profound T-cell immunosuppression [66,71,72,73].	HHV-6B encephalitis typically presents early after allogeneic HSCT as post-transplant acute limbic encephalitis, with temporal-lobe abnormalities in a subset of patients. CMV may cause ventriculoencephalitis with periventricular or ependymal abnormalities. CSF PCR or sequencing supports these diagnoses but requires clinical correlation [71,72,73].	Focal or multifocal enhancing parenchymal lesions with marked edema, a compatible *T. gondii* risk profile, *T. gondii*-specific molecular evidence, and absence of a characteristic viral syndrome increase the probability of CNS toxoplasmosis.
Bacterial or nocardial brain abscess	Relevant during neutropenia, corticosteroid therapy, transplantation, or prolonged immunosuppression; CNS nocardiosis commonly manifests as brain abscess and may accompany pulmonary or disseminated disease [66,74,75].	Fever, bacteremia, pulmonary infection, solitary or multiple abscesses with diffusion restriction, or microbiological identification from blood, respiratory material, abscess, or tissue favor bacterial or nocardial infection [74,75].	Prior *T. gondii* exposure, absent or interrupted prophylaxis, compatible CNS lesions, *T. gondii*-specific molecular evidence, and absence of microbiological evidence for bacterial or nocardial infection increase the probability of CNS toxoplasmosis.
EBV-associated CNS lymphoproliferative disease/CNS-PTLD	Relevant after allogeneic HSCT and profound immunosuppression; CNS-PTLD or EBV-associated lymphoma may mimic opportunistic CNS infection [66,76,77].	Solitary or multifocal enhancing or necrotic lesions may occur. CSF EBV DNA may support the diagnosis, but plasma EBV DNA can be low or negative in isolated CNS-PTLD; tissue demonstration of EBV-associated lymphoid proliferation may therefore be required [76,77].	A compatible *T. gondii* exposure and risk profile, *T. gondii*-specific molecular evidence, and absence of independent evidence of EBV-driven lymphoproliferative disease increase the probability of CNS toxoplasmosis.

**Note:** Anti-*T. gondii* IgG positivity indicates prior exposure and increases the pretest probability of reactivation but does not establish active CNS disease. Conversely, serological testing may be falsely negative in patients with profound B-cell depletion or hypogammaglobulinemia. All radiological and metabolic distinctions are probabilistic because diffusion, perfusion, and ^18^F-FDG uptake may overlap between toxoplasmosis, lymphoma, and other infectious or inflammatory lesions. Blood and CSF biomarkers should be interpreted in the appropriate clinical context, as their diagnostic performance varies according to the pathogen and compartment sampled. In particular, detection of HHV-6 or EBV DNA may reflect reactivation or incidental viral DNA rather than causal CNS disease, whereas negative molecular testing does not invariably exclude compartmentalized infection. Biomarker results should therefore be integrated with neuroimaging, immune status, extracerebral findings, and, when necessary, tissue diagnosis [70,71,76,77]. Histopathological confirmation should be considered when lymphoma cannot be excluded and noninvasive investigations remain inconclusive. When CSF evaluation is safe, cytology should be combined with flow cytometry. **Abbreviations:** CNS, central nervous system; CSF, cerebrospinal fluid; HSCT, hematopoietic stem cell transplantation; PCNSL, primary central nervous system lymphoma; PTLD, post-transplant lymphoproliferative disorder; PRES, posterior reversible encephalopathy syndrome; TMP–SMX, trimethoprim–sulfamethoxazole; HSV, herpes simplex virus; VZV, varicella-zoster virus; HHV-6, human herpesvirus 6; CMV, cytomegalovirus; EBV, Epstein–Barr virus.

## Data Availability

No new data were created or analyzed in this study. Data sharing is not applicable to this article.

## Supplementary Materials

The following supporting information can be downloaded at https://www.mdpi.com/article/10.3390/ijms27209063/s1.
