# Supplementary material for: Central Nervous System Toxoplasmosis Beyond HIV in Patients with Hematologic Malignancies: A Contemporary Narrative Review and Illustrative Case Synthesis
Source: Int J Mol Sci. 2026 Oct 13;27(20):9063. doi: 10.3390/ijms27209063 (PMC13705227; doi:10.3390/ijms27209063)
Supplement: Supplementary file 1 [file ijms-27-09063-s001.zip › ijms-4525432-supplementary.pdf]

Supplementary Table S1. Detailed patient-level characteristics of published reports of CNS toxoplasmosis in HIV-negative patients with hematologic malignancies.

Reporting country / year	Age/sex	Hematologic malignancy	Transplant status / timing	Serostatus / *Toxoplasma*-active prophylaxis	Blood testing	CSF testing	Key diagnostic evidence	Diagnostic category	Disease extent	Treatment / outcome	Source
USA / 2018	63/F	Chronic lymphocytic leukemia	No HSCT reported; timing NR	NR / NR	NR	NR	Brain biopsy: *T. gondii* tachyzoites	Proven	CNS-only	Pyrimethamine + sulfadiazine + leucovorin; corticosteroids for edema; clinical/radiological improvement	[10]
Ireland / 2010	63/M	Chronic lymphocytic leukemia	No HSCT reported; 8 mo after FCR	NR / NR	NR	T. gondii PCR positive	Brain biopsy: ruptured Toxoplasma cysts; concurrent JCV/PML	Proven	CNS-only	Anti-Toxoplasma treatment NR; progressive neurologic decline; death 4 wk after diagnosis	[39]
Germany / 1999	55/M	Acute T-cell leukemia, complete remission	No HSCT reported; on MTX/6-MP maintenance for 12 mo	IgG+; IgM− / NR	NR	Routine CSF unrevealing; T. gondii PCR NR	Brain biopsy: necrotizing encephalitis with *T. gondii* tachyzoites	Proven	CNS-only	Pyrimethamine + sulfadiazine; later atovaquone; slight improvement; lesions unchanged	[8]
Austria / 2026	76/M	Relapsed hairy cell leukemia	No HSCT reported; 6 mo after cladribine re-induction	IgG >300 IU/mL; IgM− / NR	NR	*T. gondii* PCR not performed	Brain histology/IHC: tachyzoites and bradyzoites	Proven	CNS-only	Pyrimethamine + sulfadiazine + folinic acid; TMP-SMX secondary prophylaxis; clinical/radiological improvement	[44]
China / 2025	5/F	Acute lymphoblastic leukemia	No HSCT reported; during chemotherapy	NR / NR	Real-time PCR negative; blood tNGS positive	CSF tNGS positive; conventional T. gondii PCR NR	*T. gondii* sequences detected by tNGS in CSF and blood	Probable	CNS-only	TMP-SMX + clindamycin; good clinical response; stable at discharge	[50]

Reporting country / year	Age/sex	Hematologic malignancy	Transplant status / timing	Serostatus / *Toxoplasma*-active prophylaxis	Blood testing	CSF testing	Key diagnostic evidence	Diagnostic category	Disease extent	Treatment / outcome	Source
USA / 2022	76/M	Relapsed/refractory IgA κ multiple myeloma	Autologous HSCT; HSCT interval NR; during active therapy	NR / NR	NR	T. gondii PCR positive (57,900 copies/mL)	Compatible CNS disease + positive CSF T. gondii PCR	Probable	CNS-only	Pyrimethamine + sulfadiazine + leucovorin; fatal basal-ganglia/intraventricular hemorrhage	[51]
Japan / 2022	76/M	Lymphoplasmacytic lymphoma, partial remission	No HSCT reported; in partial remission for 2 y	NR / NR	NR	Routine CSF findings unrevealing; T. gondii PCR NR	Autopsy: *T. gondii* cysts in brain tissue	Proven	CNS-only	Treated as CNS lymphoma; no anti-Toxoplasma therapy; death	[55]
USA / 2017	77/F	Diffuse large B-cell lymphoma, complete remission	No HSCT reported; 4 mo after remission/final R-CHOP	NR / NR	NR	T. gondii PCR positive	Post-mortem brain histology/IHC: tachyzoites and bradyzoites	Proven	CNS-only	No anti-Toxoplasma therapy; death 18 d after admission	[52]
USA / 2025	43/M	Stage IV EBV+ DLBCL-NOS with secondary HLH	No HSCT reported; after 5 cycles DA-EPOCH / during treatment course	IgG+ / NR	Initial plasma mcfDNA negative; repeat positive	T. gondii PCR negative	Brain biopsy/IHC positive for *T. gondii;* repeat plasma mcfDNA positive	Proven	CNS-only	IV then oral TMP-SMX; continued secondary prophylaxis; clinical/radiological improvement	[78]
Switzerland / 2009	56/F	CLL transformed to high-grade non-Hodgkin lymphoma	No HSCT reported; 6 wk after stopping fludarabine–cyclophosphamide / remission	NR / TMP-SMX recently discontinued	NR	Weakly positive T. gondii PCR	Autopsy/IHC: *T. gondii* in brain, spinal cord, heart and lungs	Proven	Disseminated with CNS involvement	Late pyrimethamine + sulfadiazine + clindamycin + folinic acid; death from shock/multiorgan failure	[53]
Belgium / 2021	76/M	Chronic lymphocytic leukemia	No HSCT reported; during/after obinutuzumab–chlorambucil; exact interval NR	IgG+ / NR	NR	T. gondii PCR negative	Brain biopsy: *T. gondii* pseudocysts	Proven	CNS-only	IV TMP-SMX; rapid neurological improvement; long-term outcome NR	[13]

Reporting country / year	Age/sex	Hematologic malignancy	Transplant status / timing	Serostatus / *Toxoplasma*-active prophylaxis	Blood testing	CSF testing	Key diagnostic evidence	Diagnostic category	Disease extent	Treatment / outcome	Source
Germany / 1999	61/F	Low-grade lymphoma (immunocytoma)	No HSCT reported; 6 mo after initiation of fludarabine	IgG+; IgM− / NR	NR	T. gondii PCR NR	Stereotactic brain biopsy confirmed TE	Proven	CNS-only	Pyrimethamine + clindamycin; clinical/radiological improvement	[8]
Brazil / 2000	51/M	Low-grade pleomorphic non-Hodgkin lymphoma with HAM/TSP	No HSCT reported; during intrathecal treatment; exact interval NR	NR / NR	NR	NR	Autopsy: *T. gondii* tachyzoites and cysts in brain tissue	Proven	CNS-only	Thiabendazole + allopurinol; diagnosis post mortem	[54]
Slovenia / 2016	62/F	Systemic diffuse large B-cell lymphoma	No HSCT reported; symptoms 5 d after final (8th) R-CHOP cycle	Borderline IgG+; IgM− / no Toxoplasma-active primary prophylaxis reported	NR	T. gondii PCR positive	Compatible CNS disease + positive CSF T. *gondii* PCR	Probable	CNS-only	Pyrimethamine + sulfadiazine + folinic acid; clinical/radiological improvement; mild residual cognitive deficit	[9]
Austria / 2026	61/F	Multiple myeloma, sustained remission	Autologous HSCT; interval reported inconsistently in source (4 y in case description; 26 mo in discussion); lenalidomide cycle 38 completed 1 mo before symptoms	IgG 48 IU/mL, high avidity; IgM− / NR	NR	T. gondii PCR negative; CSF T. gondii antibody borderline positive	Compatible clinical/imaging findings; no biopsy; treatment response supportive only	Possible	CNS-only	TMP-SMX → pyrimethamine + sulfadiazine + folinic acid; later lifelong TMP-SMX prophylaxis; clinical/radiological improvement	[44]
USA / 2010	70/M	Stage IV chronic lymphocytic leukemia	No HSCT reported; after FCR; exact interval NR	NR / NR	NR	NR	Autopsy: *T. gondii* in brain and pretracheal/hilar lymph nodes	Proven	Disseminated with CNS involvement	No specific anti-Toxoplasma therapy; death 2 wk after hospitalization	[42]

Reporting country / year	Age/sex	Hematologic malignancy	Transplant status / timing	Serostatus / *Toxoplasma*-active prophylaxis	Blood testing	CSF testing	Key diagnostic evidence	Diagnostic category	Disease extent	Treatment / outcome	Source
Japan / 2016	74/F	Untreated hairy cell leukemia variant	No HSCT; untreated	IgG+; IgM− / none reported	NR	*T. gondii* PCR negative; tissue PCR negative	Brain biopsy nondiagnostic; compatible imaging; treatment response supportive only	Possible	CNS-only	TMP-SMX + clindamycin → pyrimethamine + sulfadiazine + folinic acid; complete clinical/radiological response; subsequent TMP-SMX prophylaxis	[49]

Abbreviations: 6-MP, 6-mercaptopurine; CLL, chronic lymphocytic leukemia; CNS, central nervous system; CSF, cerebrospinal fluid; DA-EPOCH, dose-adjusted etoposide, prednisone, vincristine, cyclophosphamide, and doxorubicin; DLBCL, diffuse large B-cell lymphoma; DLBCL-NOS, diffuse large B-cell lymphoma, not otherwise specified; EBV, Epstein–Barr virus; F, female; FCR, fludarabine, cyclophosphamide, and rituximab; HAM/TSP, human T-cell lymphotropic virus type 1-associated myelopathy/tropical spastic paraparesis; HLH, hemophagocytic lymphohistiocytosis; HSCT, hematopoietic stem cell transplantation; IgA, immunoglobulin A; IgG, immunoglobulin G; IgM, immunoglobulin M; IHC, immunohistochemistry; IV, intravenous; JCV, JC virus; M, male; mcfDNA, microbial cell-free DNA; MTX, methotrexate; NR, not reported; PCR, polymerase chain reaction; PML, progressive multifocal leukoencephalopathy; R-CHOP, rituximab, cyclophosphamide, doxorubicin, vincristine, and prednisone; tNGS, targeted next-generation sequencing; TMP–SMX, trimethoprim–sulfamethoxazole. Time units: d, days; mo, months; wk, weeks; y, years.
